# Variable response to electric shark deterrents in bull sharks, *Carcharhinus leucas*

**DOI:** 10.1038/s41598-020-74799-y

**Published:** 2020-10-21

**Authors:** A. R. G. Gauthier, E. Chateauminois, M. G. Hoarau, J. Gadenne, E. Hoarau, S. Jaquemet, S. K. Whitmarsh, C. Huveneers

**Affiliations:** 1 Centre Sécurité Requin, 25F Avenue des Artisans, Zone Artisanale de la Pointe des Châteaux, 97436 Saint Leu, Reunion Island France; 2 Université de La Réunion UMR Entropie, 15, Avenue René Cassin - CS 92003, 97744 Saint Denis Cedex 9, Reunion Island France; 3 Southern Shark Ecology Group, College of Science and Engineering, Flinders University, Adelaide, Australia

**Keywords:** Ichthyology, Animal behaviour

## Abstract

Although relatively rare, human-shark interactions and sharks bites are increasing globally, which has led to the development of various mitigation measures. Electric shark deterrents (ESDs) have, so far, been the most effective personal deterrents, but have only been scientifically tested on one of the species most frequently responsible for shark bites, i.e. white shark (*Carcharodon carcharias*). We tested the effectiveness of five ESDs (E-Shark Force, NoShark, Rpela v2, Freedom + Surf, Freedom + Surf—Shortboard) on bull sharks, *Carcharhinus leucas*, over a period of 21 days in September 2019, in New Caledonia. Standardised bait was attached 30 cm below an experimental board that had an active ESD for up to 15 min, or until a bull shark touched the bait or the board. We compared the numbers of baits taken, numbers of passes and reactions around the board, as well as the distance between the sharks and the board among ESDs and against a control board with bait and no active ESD. The Freedom + Surf was the most effective ESD, reducing the amounts of baits taken by 42.3%, while the Rpela v2 and Freedom + Surf—Shortboard also significantly reduced the number of baits taken by 16.5% and 16.2% respectively. Mean distance between sharks and the bait was not affected by the ESDs, but the number of approaches and the proportion of reactions were both significantly higher when the Freedom + Surf was active compared to other ESDs. The effectiveness of all ESDs decreased over time, with the likelihood of the bait being taken increasing and the number of approaches and distance between sharks and the bait decreasing. Our findings show that the ability of ESDs to deter bull shark varies between products, with the Freedom + Surf resulting in the most behavioural changes, followed by the Rpela v2 and Freedom + Surf—Shortboard. However, none of the products tested completely stopped sharks from taking the bait.

## Introduction

Human-shark interactions are on the rise globally^1^. While most of these are benign and the incidence of bites is relatively low, i.e. 75–100 shark bites year^−1^, six of which result in fatalities^2,3^, public perception of the risk of shark bites and ensuing fatality is much higher than reality^4,5^. The frequent negative framing by mass and social media might have contributed to accentuated public anxiety and negative perception about sharks^6,7^, which in turns can affect local economies^1,8^. Such high level of public concern has led to major investments from governments and private companies to the development of new strategies to mitigate the potential risks of shark encounters^1,9–11^.

In recent years, many commercially-available personal shark deterrents have been developed to mitigate shark bite risk and are now widely used. Among these, a range of electric shark deterrents (ESDs) were created, aiming to overwhelm the electrosensory system of sharks. The Shark Shield technology that powers Ocean Guardian products has been tested several times, purportedly repelling sharks in 60%^12^ and 89%^13^ of trials for their Freedom + Surf and Freedom7 products respectively (https://ocean-guardian.com/). A more recent study showed that the Scuba7 reduced the risk of bites from *Carcharhinus melanopterus* by 67%^14^. The Rpela, an ESD for surfers requiring some experience in shaping boards or using fibreglass, did not reduce the probability of a bait being taken^12^. However, an updated version released following Huveneers et al. (2018)—Rpela v2—reduced the number of shark bites by 66%^15^ (www.rpela.com). The E-Shark Force and the NoShark are deterrents worn around the ankle or wrist, available with an optional leash (www.bluvand.com; www.e-sharkforce.com). The effectiveness of a previous version of the NoShark, the Electronic Shark Deterrent System (ESDS), showed no reduction in the risk of a shark bite because of its short effective range^16^.

Elasmobranch ability to detect minute electromagnetic fields is species-specific and depend on the species morphology, habitat, or their foraging strategies^17^. Visual predators such as white sharks, *Carcharhodon carcharias*, or scavengers like tiger sharks, *Galeocerdo cuvier*, typically have fewer electroreceptors than species like bull sharks, *Carcharhinus leucas*, which likely results in a lower reliance of the electrosensory system in white and tiger sharks^17^. In elasmobranchs, electromagnetic fields are detected by the ampullae of Lorenzini located in the head of sharks and in the head and pectoral fins of skates and rays^18^. Interspecific variations in the number, distribution, and sensitivity of these ampullae may have an impact on elasmobranch tolerance to the electric pulses emitted by ESDs. Previous studies have shown that the threshold at which sharks are behaviourally affected varies across species and can ranges from 3 V/m in bull sharks to 18.5 V/m in scalloped hammerheads, *Sphyrna lewini*^10,19^. As of today, most ESDs have either not been tested independently and scientifically or were tested primarily on one species, i.e. the white shark^12,13,15,16,20,21^. The efficacy of these devices on other potentially dangerous shark species such as bull sharks or tiger sharks is therefore lacking.

There is evidence that sharks exposed repeatedly to electromagnetic fields may learn to tolerate them^22,23^. The potential for shark to become habituated to the electric fields and become less responsive to ESDs have been frequently discussed^12,13,16^. Studies on the effects of magnets or electric fields show that shark response can decrease through regular exposure^22,23^. Observations by Kempster et al. (2016) showed that white sharks displayed evidence of habituation throughout their study, with specific individuals getting closer to an active Freedom7 deterrent at each pass. However, Huveneers et al. (2018) and Egeberg et al. (2019) showed little temporal effects. This discrepancy warrants further investigation of the effects of frequent exposure to ESDs.

In this study, we aimed to assess the efficacy of several ESDs to better understand the ability of these ESDs to protect ocean users from shark bites and to assess the effects of repeated exposure to ESD electric pulses on shark behaviour. Our study was the first to investigate the effectiveness of five personal electric shark deterrents, the E-Shark Force, NoShark, Rpela v2.0, Freedom + Surf, and Freedom + Surf—Shortboard on bull sharks, one of the species most implied in shark bites around the world^1^.

## Methods

### Study species and site

The experiment took place over 21 days in September 2019 in the Nouville harbour in New Caledonia (22° 16′ 01.6′′ S 166° 25′ 25.6′′ E), an area selected for its relatively high abundance of resident *C. leucas* (*Pers. Comm.* Province Sud/IRD) that were fed fish scraps by commercial fishers unloading their catch. Since March 2017, strict regulations are now in place, prohibiting feeding and discharge of fish blood at that site. All the methods were performed in accordance with the relevant guidelines and regulations for experimenting on live vertebrates (bull sharks; Flinders University Animal Ethics Committee approval E446) and with the Province Sud’s approval (permit no: 2844-2019/ARR/DENV).

### Shark deterrent set-up

**Figure 1:**
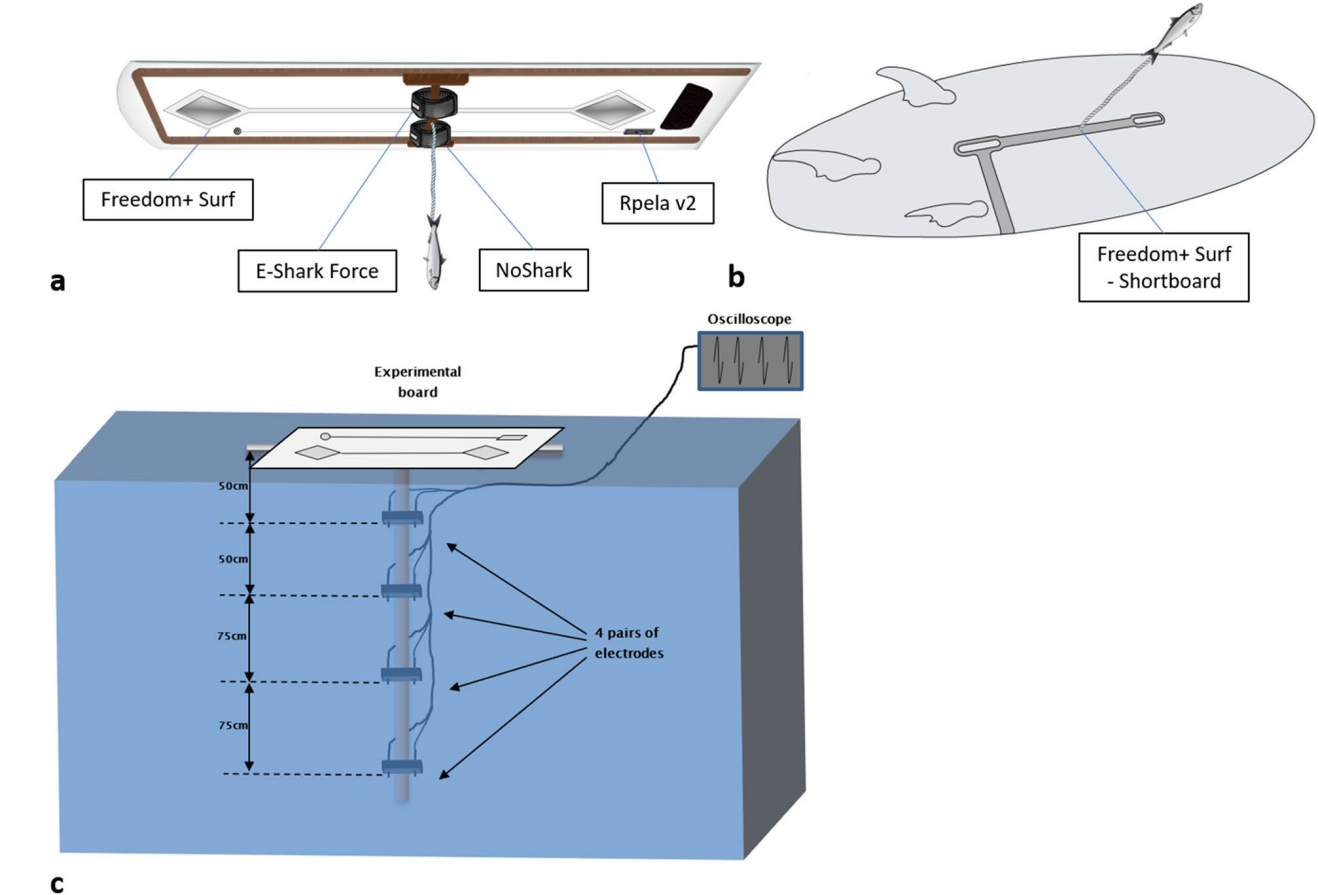
Experimental set ups. Illustrations of the two experimental boards ((**a**) 130 × 33 cm, (**b**) 180 × 46 cm) with the five deterrents tested and bait attached. (**c**) Experimental set up for the electric field modelling. Illustrations by Rachel Flavigné, Centre Sécurité Requin.

We first tested four commercially-available electric shark deterrents: the E-Shark Force, the NoShark, the Rpela v2, and the Freedom + Surf, using a custom-built surfboard replica. The experimental board was 130 cm × 33 cm, made of polymethyl methacrylate and strengthened with wood on the sides and in the middle of the board where the bait was attached (Fig. 1a). The deterrents were either placed as per manufacturer instructions or in the middle of the board mimicking how a person would wear it, e.g. someone wearing the E-Shark force on her/his ankle and sitting on the surfboard while waiting for a wave. The same board was used to test all four deterrents, with one deterrent active (or no deterrents active for control) for each trial. Non-active deterrents were either turned off (Freedom + Surf), or replaced by replicas (E-Shark Force, NoShark, and Rpela v2).

We subsequently tested an additional deterrent, the Freedom + Surf—Shortboard, installed on a surfboard (180 cm × 46 cm, Fig. 1b). The same surfboard served as a control, with the Freedom + Surf—Shortboard turned off. Because of technological difficulties with the device, the tests of the Freedom + Surf—Shortboard could not occur at the same time as the other deterrents, and only started on Day 12 of the trials. Testing of the Freedom + Surf—Shortboard followed the same protocol as the other deterrents, also with an attached bait.

### Experiment

For each trial, a dead sardine, *Sardina pilchardus* (*ca*. 20 cm long and 50 g), was attached by a 30 cm rope below the experimental board. Each trial series consisted of a trial for each deterrent and the control (five trials), in random order, and each trial lasted 15 min, or until the bait was either touched, taken, or the board was touched. If the board lasted for 15 min without being touched, or without the bait being touched or taken, the trial was considered a success. Trials were only started after at least two sharks were observed within the testing area. Trials were removed from the analysis if no sharks were recorded around the experimental board or if none approached the board. Five to nine trial series were performed between 07:00 and 16:00 on each of the 18 days of the study. Trials were performed consecutively, with a few minutes between each trial to change the active ESD and replace the bait. Each trial was recorded using a stereo-video unit consisting of two GoPro Hero 4 mounted in secure custom-built housings (SeaGIS Pty Ltd, Victoria, Australia) angled 8 degrees inward and set 76 cm apart. The stereo-video unit was handheld from the pier the experiments were conducted from, using a pole attached to the unit and placed about 50 cm underwater and 2–3 m from the experimental board. The use of the stereo-video unit allowed for accurate measurements of size of sharks and distance to bait to be recorded^24^. The cameras were calibrated using SeaGIS EventMeasure. We measured the outcome of each trial, as well as the number of passes and reactions exhibited by sharks. We measured the minimum distance between the shark and the experimental set up for each pass when possible (when both the snout and bait were clearly visible). The distance was measured between the tip of the snout, and either the top of the bait or the experimental board, whichever the shark was closest to. For each pass, we attempted to identify individual sharks using a combination of factors, such as scars, markings, or fin shape.

### Video processing

The coder was ‘blind’, as he did not participate in the experiment and did not know which deterrent was tested in each video. We used Huveneers et al. (2018)’s terminology to describe and code the sharks’ behaviour. Briefly, a *pass* characterised a shark swimming towards the experimental set up (each time a shark veered away from the board and swam back we classified it as a new pass). *Shark ID* referred to the individual sharks that we identified by body markings. *Distance* is the minimum distance between the shark and either the bait or the board, whichever was closest, for each pass. *Intent* represented the shark’s motivation when approaching the bait, classified as either “Low”, “Medium”, or “High” using a combination of factors including shark swimming direction in relation to the bait, swimming speed, and acceleration. Low: shark moving slowly, not approaching in the direction of the bait and without acceleration; Medium: shark slowly moving towards the bait without acceleration; High: shark approaching the bait at speed or accelerating. Only the passes with an *Intent* classified as either “Medium” or “High” were kept for the analysis. Finally, a *reaction* was recorded when the coder observed a behavioural reaction towards the experimental setup, e.g. tail flick, muscle spasm, head shake, visible nictitating membrane, fast direction change.

### Statistical analysis

We investigated the effects of all five ESDs on the following response variables: whether the bait was taken, distance to the bait, number of approaches, and whether the sharks reacted to the deterrents. We first tested the effects of all five ESDs on all response variables using the complete dataset with a generalised linear model (GLM), with *Deterrent* and *Trial series* as fixed effects. We followed this analysis with a generalised linear mixed-effects model (GLMM), with *Shark ID* coded as a random effect, *Deterrents* and *Trial series* as fixed effects, and removing all the unidentified sharks from the dataset. Due to poor visibility, sharks could only be identified in 47% of passes, so using *Shark ID* as a random factor to account for pseudo-replication reduced the dataset. While using *Shark ID* as a random factor improves model fit, it leads to 53% of passes being removed from the analysis. Hence, we present the results from both the GLM and GLMM to demonstrate the effect of accounting for the behaviour of individual sharks. Models were fitted with a gaussian distribution for *Distance* and *Number of approaches*, and a binomial distribution with logit link for the likelihood of the bait/board being touched/taken and the likelihood of a reaction. The *Number of approaches* was log transformed and scaled prior to fitting the GLM and GLMM. We included the interaction between trial and deterrent to account for potential temporal effects. We ran all models for all possible combinations of factors and compared their relative probability using Akaike’s information criterion corrected for small sample size (AIC_c_)^25^.

Additionally, we compared the proportion of times the trials ended up in success, and the proportion of reactions exhibited by sharks between deterrents using the ‘minlike’ method two-sided Poisson exact test from the *exactci* R package^26^. All statistical analyses were conducted with the RStudio software, version 1.2.5033. All figures represent the full dataset used for the GLM, combining both identified and unidentified sharks.

### Electric field modelling

We modelled the electric field propagation of the E-Shark Force, NoShark, Rpela v2, and Freedom + Surf in the water following the methodology previously used to test these devices in a controlled environment^27^. The Freedom + Surf—Shortboard was excluded from these tests as the device was not functional at the time of testing. The experimental set up was adapted to simultaneously measure the electric potential differences across four electrode pairs. The electrode pairs were fixed on a T-shaped polyvinyl chloride frame and placed vertically at a distance of 50, 100, 175, and 250 cm from the centre of the electrodes of each ESD and connected to a digital oscilloscope that allowed for the simultaneous measurement of the electric potential around the four electrodes pairs (Fig. 1c). All the electrodes were orientated in the same direction as the direction of propagation of the electric fields emitted by the ESDs, based on the in vitro tests previously performed^27^.

The electric field was modelled as a dielectric dipole, where each conductive electrode was considered a point load with a charge of q and −q. The distance from each electrode is adapted to the modelized ESD to account for the differences in electrode placement. Each charge delivers a potential scalar field V_q_ (Eq. (1)):

1 $${V}_{q}(\mathrm{d})=\frac{q}{4\pi {\varepsilon }_{0}{\varepsilon }_{r}}\frac{1}{d}$$

where ${\varepsilon }_{0}$ is the permittivity of the empty space; ${\varepsilon }_{r}$ the relative permittivity of the considered material; and d the distance from the charge q.

The effect of the water surface on the field was taken into account using the method of image charges^14^. This method states that, for a given distribution of charges, the solution of the Poisson equation is unique. Using this principle, we can add some fictive charges to simulate the presence of a non-conductive field (air) to cancel the electric field flux through the water surface. Therefore, some fictive charges were added to simulate the water surface proximity. The total electric field delivered by the dipole [q,-q] is then calculated by adding the potential scalar field of each electrode (fictive and non-fictive), and applying the classic electromagnetism results derived from the Poisson equation (Eq. (2)):

2 $$\overrightarrow{E}(\mathrm{r})=-\mathrm{grad }\left({V}_{tot}\right)$$

Hence we know the shape of the electric field, and the model parameters of each ESD ($\mathrm{K}=\frac{q}{4\pi {\varepsilon }_{0}{\varepsilon }_{r}}$) can be determined by dichotomous principle to obtain a theoretical curve as close to the measured values as possible using the least square method. This model is then used to calculate the maximum depth where the electric fields reach a 3 V/m intensity, which is the supposed minimum intensity at which bull sharks are effectively repelled by electric deterrents^10^. Additionally, we performed a tri-dimensional analysis of the isosurfaces equal to an electric field of 3 V/m using the previously calculated theoretical model. All these analyses were performed using PyCharm 2020.1.

## Results

We did 536 trials from which we removed 18 trials due to technological issues at the time of deployment or because no sharks interacted with the board (Control: 75 trials, E-Shark Force: 74, NoShark: 78, Rpela v2: 79, Freedom + Surf: 78, Freedom + Surf—Shortboard (Control): 68, Freedom + Surf—Shortboard: 68). Out of the remaining 518 trials, we recorded 1394 passes from a total of 29 individual sharks (657 and 737 passes from identified and unidentified sharks, respectively) ranging from 1.9 to 2.9 m TL. Sharks could not be identified for 53% of the passes because of low visibility of ~ 2–3 m (max ~ 4 m). The mean (± standard error) distance between shark and bait, not including the passes where sharks successfully got the bait or touched the board, was 40.1 ± 1.4 cm (0.0–351.0 cm, n = 1179). Sharks approached the board for an average of 2.6 ± 0.1 (range 1–24) passes per trial. Individual sharks approached the board for 22.9 ± 9.4 passes (range 1–227), and 15.6 ± 6.4 (range 1–153) trials. We recorded 36 passes with “Low” intent, 292 with “Medium” intent, and 1066 with “High” intent.

**Figure 2:**
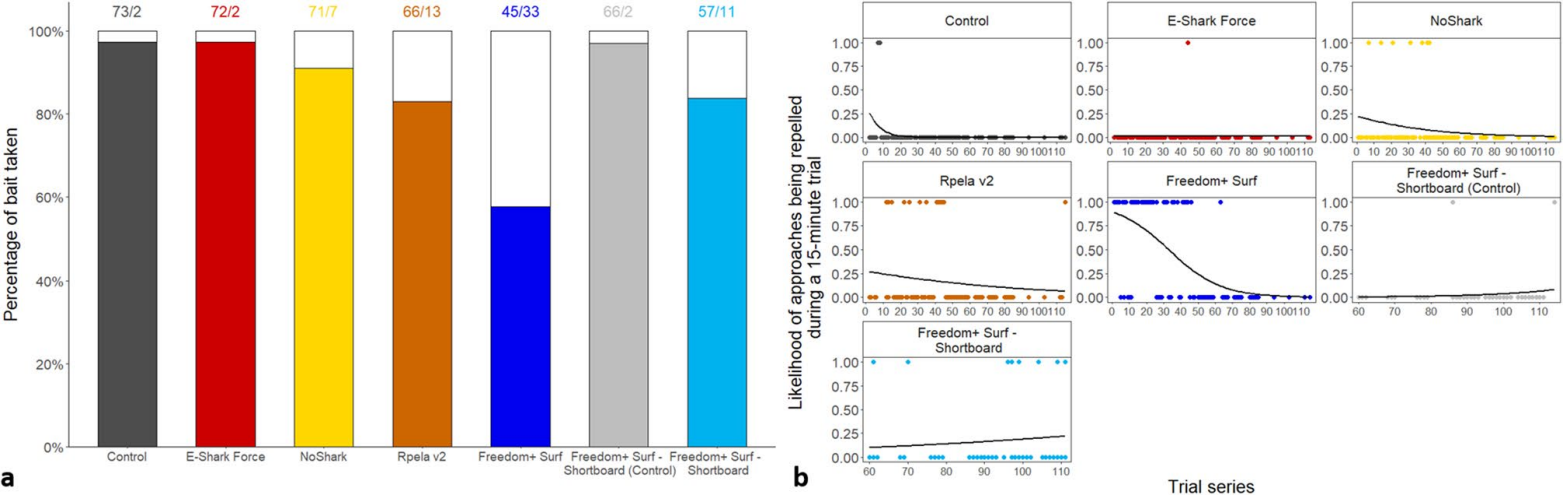
Effects of ESDs on the percentage of trials a bull shark took/touched the bait/board. (**a**) Effects of ESDs on the percentage of baits taken by bull sharks during trials. White bars represent trials when the board or bait were not touched or taken. Numbers above the bars represent the number of trials with board or bait touched/taken vs. not touched/taken. (**b**) Logistic regression of the likelihood of approaches being repelled during a 15-min trial over time.

**Figure 3:**
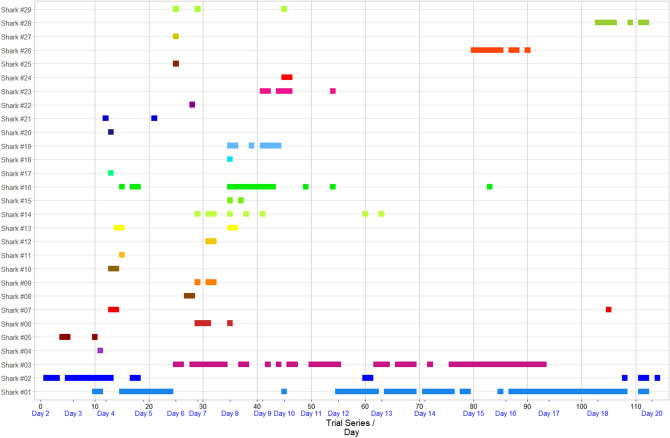
Abacus plot showing the frequency of interactions from the 29 sharks that could be identified during the study.

**Figure 4:**
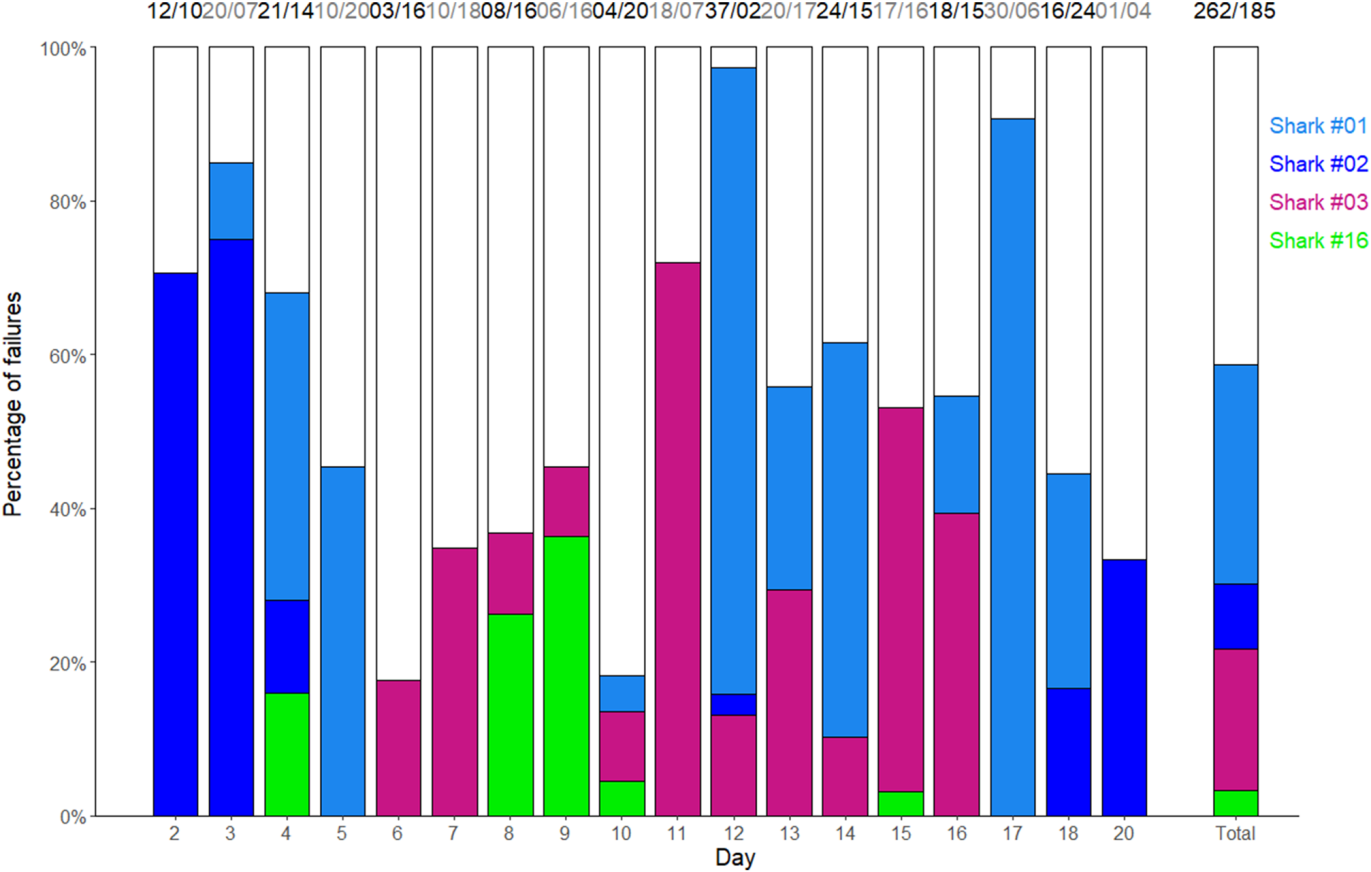
Percentage of bait/board taken/touched by the four sharks that interacted with the board in 15 trials or more. Numbers above the bars represent the number of times the bait/board was taken/touched by these four sharks/unidentified sharks. Light blue: Shark #01, dark blue: Shark #02, violet: Shark #03, green: Shark #16, white: unidentified sharks.

When no deterrents were active (i.e., on the control board), sharks touched or bit the bait or the board (hereafter referred to as an ‘interaction’) 97.3% of the time. The deterrents had various effects, with the percentage of times sharks interacted with the bait ranging from 57.7% (Freedom + Surf) to 97.3% (E-Shark Force) (Fig. 2a), with the likelihood of sharks being repelled during a 15-min trial generally decreasing overtime for the Freedom + Surf, Rpela v2, and NoShark (Fig. 2b). The Freedom + Surf, Rpela v2, and Freedom + Surf—Shortboard all significantly reduced the percentage of trials ending with an interaction (Poisson exact test: *p* < 0.01 for all three, *p* = 1.00 for the E-Shark Force, *p* = 0.18 for the NoShark). The top-ranked model included deterrent and trial series for both the GLM and GLMM (wAIC_c_ = 0.99 for both; Tables 1, 2). The Freedom + Surf had the largest positive effect on the number of baits taken compared to the other deterrents (Tables S1, S2). Four sharks participated in more than 15 trials: Shark #01 (153 trials), #03 (115 trials), #02 (47 trials), and #16 (25 trials) (Fig. 3, S1–S3). Out of the 447 trials where the bait/board were touched or taken, Shark #01 was responsible for 127 (28.4%), Shark #03 for 82 (18.3%), Shark #02 for 38 (8.5%), and Shark #16 for 15 (3.4%); 58.6% of all failed trials (Fig. 4). The top-ranked model still included deterrent and trial series after removing Sharks #01, #02, #03, and #16 from the dataset (Table S3).

**Table 1:** Summary of the different generalized linear models tested to estimate the effects of deterrents.

Model	k	AICc	ΔAICc	wAICc	R
**(a) Probability of the board or bait being touched or bitten by bull sharks (binomial error distribution)**					
Bait ∼ deterrent + trial + deterrent × trial	14	313.02	0.00	0.99	0.29
Bait ∼ deterrent + trial	8	321.54	8.52	0.01	0.24
Bait ∼ deterrent	7	344.55	31.53	< 0.01	0.16
Bait ∼ trial	2	388.30	75.28	< 0.01	0.05
Bait ∼ 1 (intercept-only)	1	408.27	95.25	< 0.01	0.00
**(b) Number of reactions by bull sharks after exposure to the board (binomial error distribution)**					
Reactions ∼ deterrent	7	1624.00	0.00	0.72	0.18
Reactions ∼ deterrent + trial	8	1626.00	2.00	0.27	0.18
Reactions ∼ deterrent + trial + deterrent × trial	14	1632.20	8.20	0.01	0.19
Reactions ∼ trial	2	1873.00	249.00	< 0.01	0.01
Reactions ∼ 1 (intercept-only)	1	1878.40	254.40	< 0.01	0.00
**(c) Distance between bull shark and the board (Gaussian error distribution)**					
Distance ∼ deterrent + trial + deterrent × trial	15	708.18	0.00	0.99	0.12
Distance ∼ deterrent + trial	9	716.85	8.67	0.01	0.10
Distance ∼ trial	8	728.01	19.83	< 0.01	0.07
Distance ∼ deterrent	3	765.77	57.59	< 0.01	0.03
Distance ∼ 1 (intercept-only)	2	778.02	69.84	< 0.01	0.00
**(d) Number of passes by bull sharks towards the board (Gaussian error distribution (log link))**					
Passes ∼ deterrent + trial	9	− 37.95	0.00	0.86	0.11
Passes ∼ deterrent + trial + deterrent × trial	15	− 41.65	3.70	0.14	0.12
Passes ∼ deterrent	8	− 9.99	27.96	< 0.01	0.07
Passes ∼ trial	3	− 3.28	34.67	< 0.01	0.04
Passes ∼ 1 (intercept-only)	2	28.54	66.49	< 0.01	0.00

*k* number of model parameters, *AIC*_*c*_ Akaike’s information criterion corrected for small sample size, *ΔAIC*_*c*_ difference in *AIC*_*c*_ between the current and top-ranked model, *wAIC*_*c*_ model probability, *R* pseudo-R^2^.

**Table 2:** Summary of the different generalized linear mixed models tested to estimate models estimating the effects of deterrents.

Model	k	AICc	ΔAICc	wAICc	Rm
**(a) Probability of the board or bait being touched or bitten by bull sharks (binomial error distribution (logit link))**					
Bait ∼ deterrent + trial	9	126.34	0	0.99	97.2
Bait ∼ deterrent	8	137.83	11.5	< 0.01	96
Bait ∼ trial	3	155.88	29.54	< 0.01	37.5
Bait ∼ 1 (intercept-only)	2	167.07	40.74	< 0.01	21.8
**(b) Number of reactions by bull sharks after exposure to the board (binomial error distribution (logit link))**					
Reactions ∼ deterrent + trial	9	749.37	0.00	0dis.52	37.8
Reactions ∼ deterrent	8	749.56	0.19	0.48	37.4
Reactions ∼ trial	3	914.37	164.99	< 0.01	5.6
Reactions ∼ 1 (intercept-only)	2	916.71	167.33	< 0.01	4.8
**(c) Distance between bull shark and the board (Gaussian error distribution (log link))**					
Distance ∼ deterrent + trial	10	315.55	0	0.76	20.3
Distance ∼ trial	4	320.91	2.36	0.24	17.5
Distance ∼ deterrent	9	339.8	21.24	< 0.01	13.4
Distance ∼ 1 (intercept-only)	3	346.99	28.44	< 0.01	15
**(d) Number of passes by bull sharks towards the board (Gaussian error distribution (log link))**					
Passes ∼ deterrent + trial	10	− 175.92	0.00	0.95	10.5
Passes ∼ trial	4	− 169.8	6.11	0.05	6.4
Passes ∼ deterrent	9	− 161.66	14.27	< 0.01	5.2
Passes ∼ 1 (intercept-only)	3	− 149.63	26.29	< 0.01	0

All models include Shark ID as a random effect.

*k* number of model parameters, *AIC*_*c*_ Akaike’s information criterion corrected for small sample size, *ΔAIC*_*c*_ difference in *AIC*_*c*_ between the current and top-ranked model, *wAIC*_*c*_ model probability, *R*_m_ marginal (fixed effect) R^2^.

**Figure 5:**
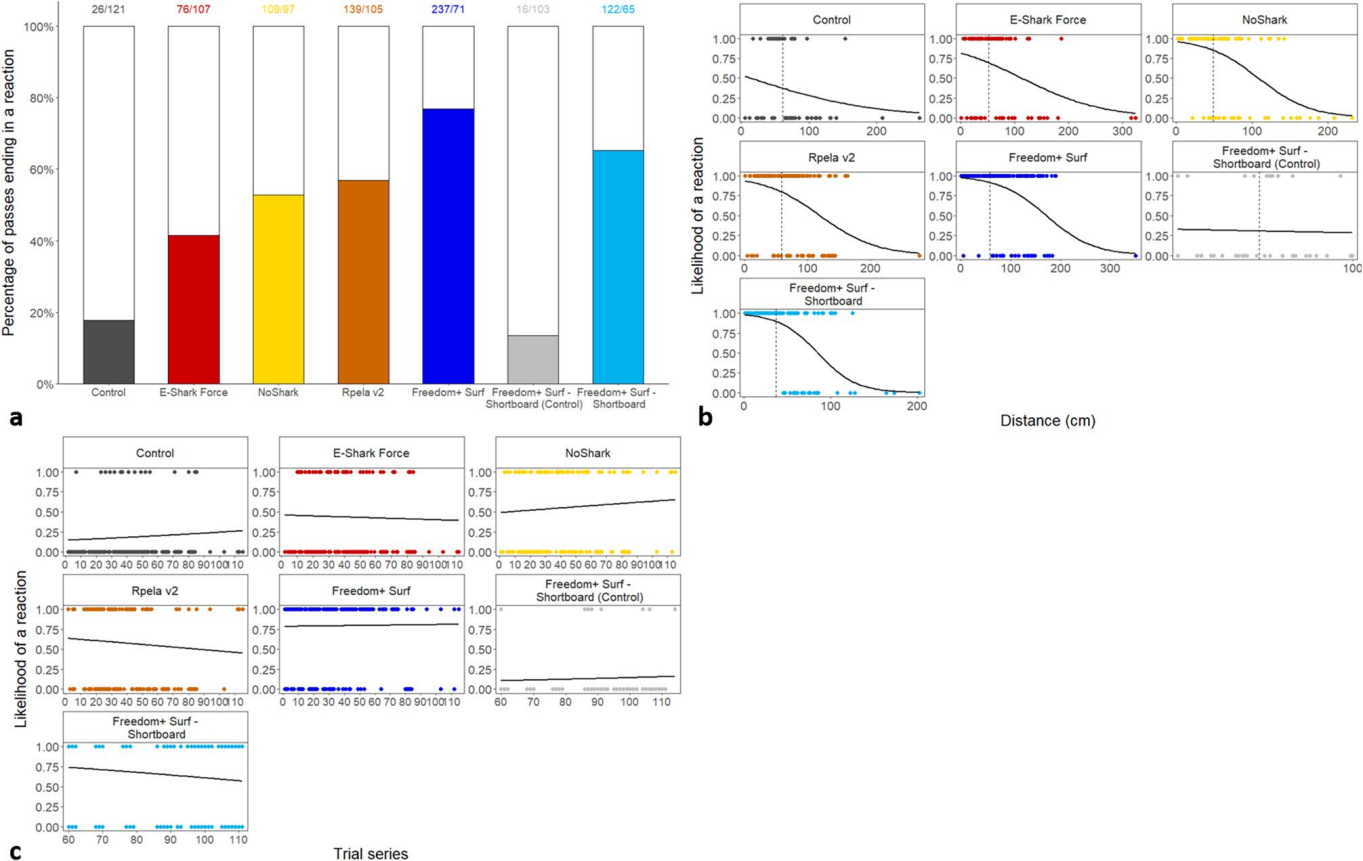
Effects of ESDs on the chances of a bull shark exhibiting a reaction during an approach. (**a**) Effects of ESDs on the percentage of reactions exhibited by bull sharks during trials. White bars represent passes with no reactions. Numbers above the bars represent the number of passes with or without reactions. (**b**) Logistic regression of the likelihood of a shark reacting to an ESD depending on the distance at which they approached the bait. Dashed lines represent the mean distance at which sharks approached the experimental board. (**c**) Logistic regression of the likelihood of a shark reacting to an ESD over time.

We observed 725 reactions, which occurred at an average distance of 44.3 ± 15.7 cm (0–192.0 cm) and which were more likely to occur when a deterrent was active (Fig. 5a). The percentage of passes ending in a reaction increased with all deterrents (Poisson exact test: *p* < 0.01), but varied from 41.5% for the E-Shark Force to 77.0% for the Freedom + Surf *vs*. 17.7% for the control boards (Fig. 5a). The top-ranked model included deterrent for the GLM (wAIC_c_ = 0.72), and deterrent and trial series for the GLMM (wAIC_c_ = 0.52) (Tables 1, 2). However, trial likely did not strongly influence the likelihood of a reaction because the next-ranked model did not include trial, had a slightly lower wAIC_c_ (0.48), and the trial coefficient was small (− 0.006) (Tables 2, S2). The Freedom + Surf had the largest positive effect on the likelihood of reactions compared to the other deterrents (Tables S1, S2). The likelihood of a reaction decreased with distance to the board in all deterrents (Fig. 5b, Table S3) but was relatively stable over time (Fig. 5c).

**Figure 6:**
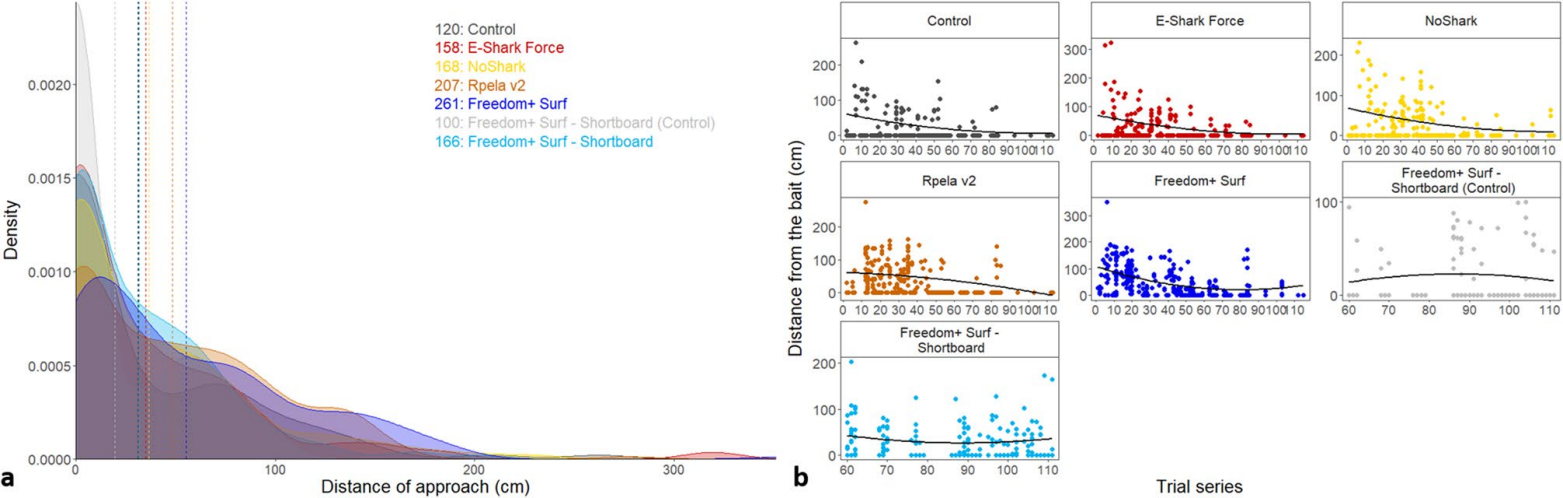
Effects of ESDs on the distance at which bull sharks approached the bait. (**a**) Density distribution of the distance between bull sharks and the bait. Dash lines represent the means for each deterrent. Coloured numbers represent the number of passes from which the density distributions were calculated. Dark grey: Control, red: E-Shark Force, yellow: NoShark, Orange: Rpela v2, dark blue: Freedom + Surf, light grey: Freedom + Surf—Shortboard (Control), light blue: Freedom + Surf—Shortboard. (**b**) Regression of the distance at which bull sharks approached the bait over time.

The mean distance at which sharks approached the board ranged from 35.9 ± 4.2 cm for the E-Shark Force, to 55.1 ± 3.4 cm for the Freedom + Surf, compared to 31.7 ± 4.3 cm for the Control. Sharks approached the Freedom + Surf—Shortboard board at a distance of 20.1 ± 3.0 cm for the control, and 31.6 ± 2.9 cm when the device was active (Fig. 6a). The distance at which sharks approached the board decreased over time for all deterrents but the Freedom + Surf—Shortboard and its control board (Fig. 6b). The top-ranked model (wAIC_c_ = 0.95) included deterrent and trial series for both the GLM and GLMM (wAIC_c_ = 0.99 and 0.76 respectively) (Table 1). In the GLM analysis, the NoShark had the largest positive effect on the distance at which sharks approached the board (0.02) compared to the other deterrents (Table S1), whereas in the GLMM, the Freedom + Surf—Shortboard (Control) had the largest positive effect compared to the other variables (Table S2).

**Figure 7:**
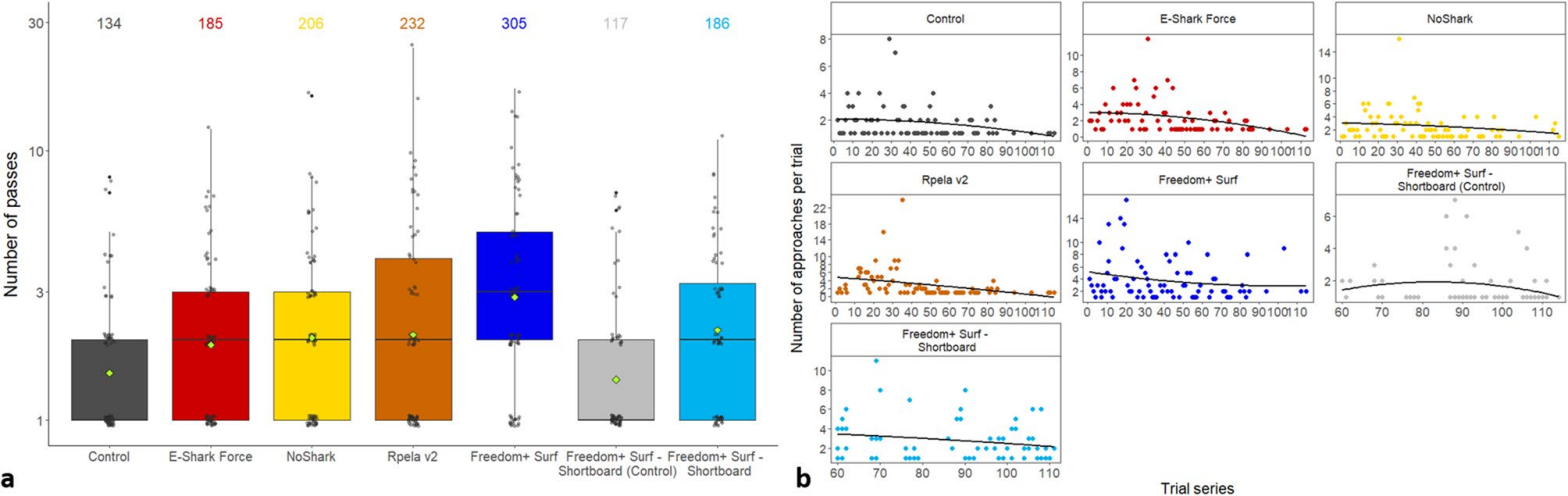
Effects of ESDs on the number of times bull sharks approached the bait. (**a**) Number of passes trial-1 shark-1 during 15-min trials (grey circles with small ‘jittering’ to improve readability). Median values are indicated by the horizontal bar, length of the box is the interquartile range, whiskers represent quartiles, circles are data, and green diamond is the mean. Y-axis shown on the log10 scale. Numbers indicate the total number of passes. (**b**) Regression of the number of approaches over time.

The mean number of passes was highest when the Freedom + Surf was active (3.9 ± 0.4), but the presence of an active deterrent resulted in a higher number of passes for all other deterrents (2.5 ± 0.2–3.1 ± 0.4) compared to the two control boards (1.7 ± 0.2–1.8 ± 0.2; Fig. 7a). The number of approaches generally decreased over time (Fig. 7b). The top-ranked model included deterrent and trial series for both the GLM and GLMM (wAIC_c_ = 0.86 and 0.95 respectively; Tables 1, 2). The Freedom + Surf had the largest effect on number of passes compared to the other variables (Tables S1, S2).

**Figure 8:**
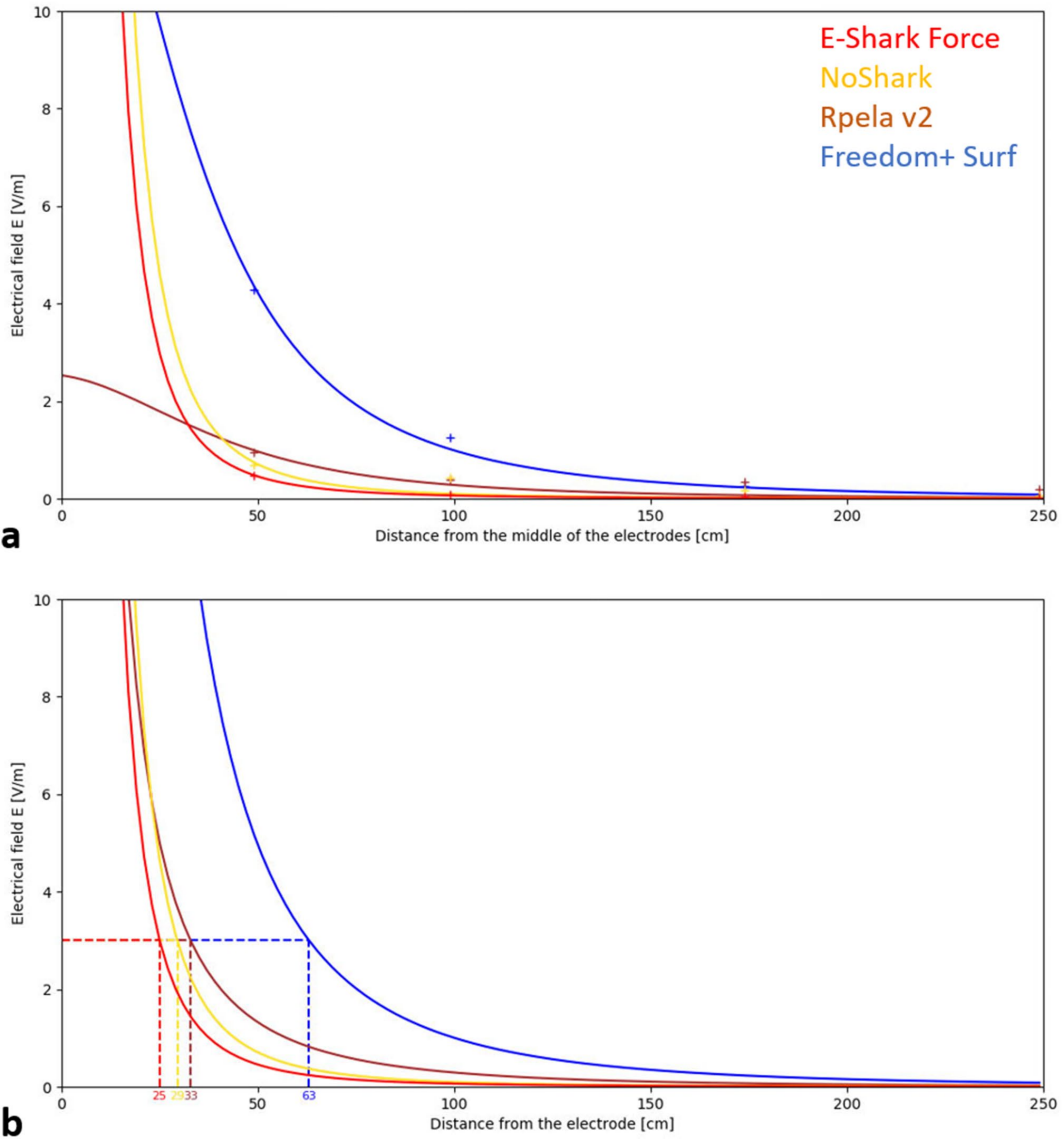
Theoretical electric field strength for each ESD. (**a**) Theoretical electric field strength for each ESD between the two ESD electrodes, calibrated by the in vivo measurements (“+” marks). (**b**) Distance measurement for the 3 V/m threshold, directly below the ESD electrodes. Red: E-Shark Force, yellow: NoShark, brown: Rpela v2, blue: Freedom + Surf.

The intensity of the electric pulses emitted by the ESDs varied in strength, the strongest being the Freedom + Surf at a distance of 50 and 100 cm from the electrodes (4.28 and 1.25 V/m respectively), and the Rpela v2 at a distance of 175 and 250 cm (0.35 and 0.19 V/m respectively; Table 3). A theoretical model was derived for each deterrent from these values (Fig. 8a). The theoretical distance at which the electric fields produced by each ESD reach an intensity of 3 V/m was ~ 30 cm for the E-Shark Force, NoShark, and Rpela v2, and 63 cm for the Freedom + Surf (Fig. 8b).

**Table 3:** Intensity of the electric fields for each ESD. Measurements were taken along a vertical segment from the centre of the electrodes.

Distance (cm)	Electric fields E (V/m)			
	E-Shark force	NoShark	Rpela v2	Freedom + Surf
50	0.48	0.69	0.95	4.28
100	0.08	0.43	0.38	1.25
175	0.04	0.17	0.35	0.27
250	0.02	0.08	0.19	0.02

**Figure 9:**
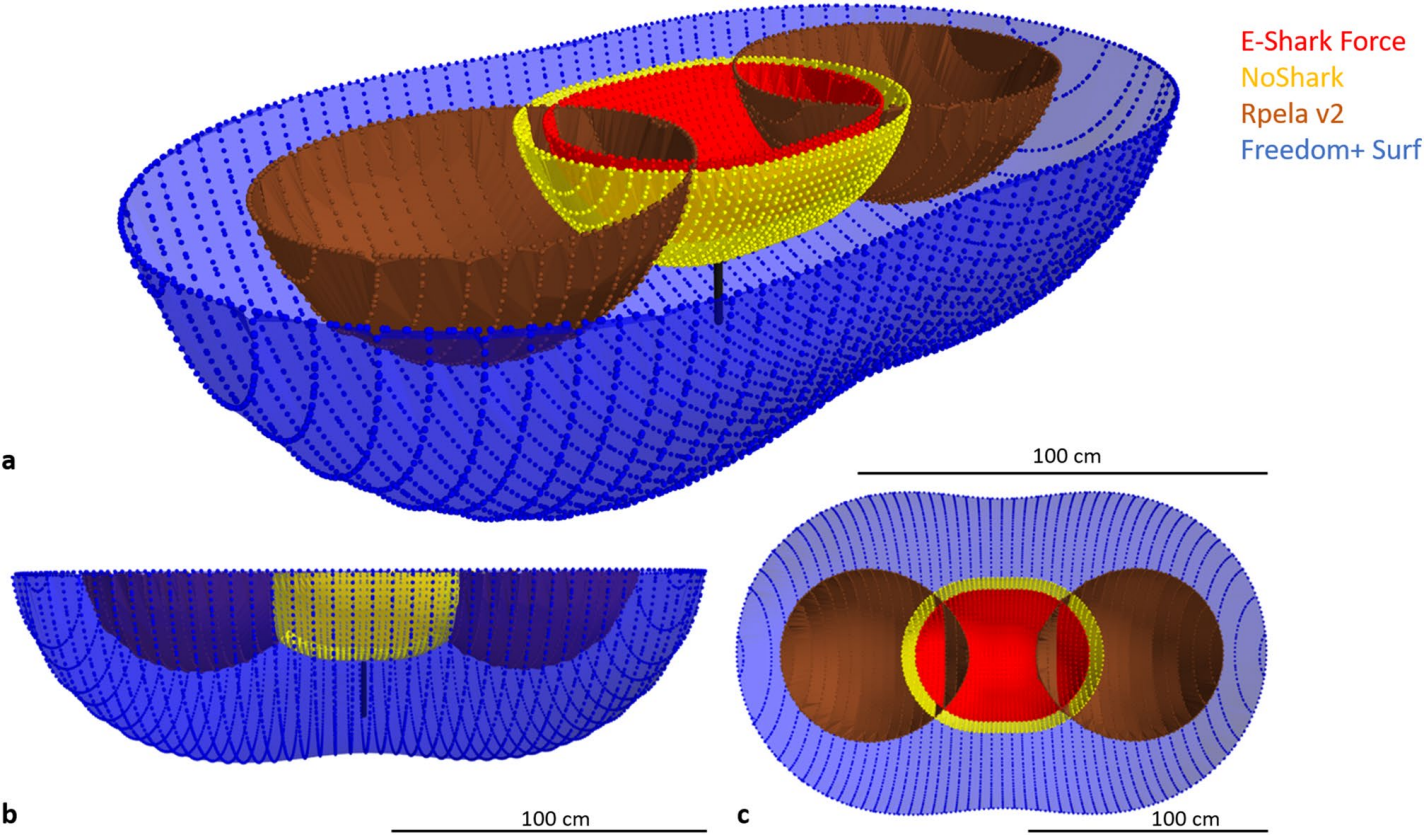
Tri-dimensional representation of a constant field of 3 V/m for each ESD. The black line represents the theorical position of the bait during the experimentation (~ 30 cm below the surface). Red: E-Shark Force, yellow: NoShark, brown: Rpela v2, blue: Freedom + Surf. Scale bars = 100 cm.

The calculations allowed for a tri-dimensional representation of the theoretical volume each ESD covers for a constant of E = 3 V/m (Fig. 9). A 3 V/m isosurface for both the E-Shark Force and NoShark appear as a hemi-ellipsoid with a slight bottleneck in the middle. The fields respectively extend up to 72 and 84 cm horizontally, 56 and 64 cm in width, and 25 and 29 cm in depth. The shapes of the Rpela v2 fields are quasi-hemispherical around each electrode. Each of the quasi-hemisphere extends up to 78 cm long, 72 cm wide, and 33 cm deep and do not overlap in-between the electrodes. A 3 V/m isosurface for the Freedom + Surf appears as a hemi-ellipsoid with a slight bottleneck in-between the two electrodes. The field extends up to 220 cm horizontally, 134 cm in width, and 63 cm in depth directly below the electrodes (Fig. 9).

## Discussion

Our study shows that the ability of electric shark deterrents to reduce human-shark interactions extend beyond white sharks and that some products can affect the behaviour of and bite risk from bull sharks. The behavioural response of bull sharks was, however, highly variable across deterrents, and likely affected by the electrode placement and strength of the electric field produced. In-situ measurements and theoretical modelling of the electric field propagation in seawater supports our observation showing the Freedom + Surf to be the most efficient deterrent of the tested models. While the electric fields produced by the products tested may vary across a range of characteristics (e.g. voltage, frequency, pulse duration, type of pulse), our study reinforces that field propagation is a key factor in deterring sharks and reducing shark bite risks. The efficacy of these deterrents appears to decrease over time, suggesting some degree of habituation of the sharks more frequently exposed to the different electric fields.

The behavioural tests found the Freedom + Surf to be the most efficient ESD. A fact corroborated by the electric field modelling where a clear hierarchy is established in the size of the electric fields produced by ESDs, with the Freedom + Surf having the largest volume of coverage, followed by the Rpela v2, NoShark, and E-Shark Force. The Freedom + Surf is the only deterrent for which an isosurface of 3 V/m, the maximum theoretical field strength that bull sharks can tolerate^10^, completely covers the bait. However, its efficacy in our study appears to be lower than in Huveneers et al. (2018), with 42.3% of our trials ending in a success, as opposed to 56% for Huveneers et al. (2018). This discrepancy may be caused by several factors, such as the possible competition between sharks, with sometimes up to six or seven sharks present during a single trial, competing for a small reward^28,29^. The high number of bull sharks present on site may be an additional motivator to ignore, or better tolerate, the electric fields produced by the deterrents. Interestingly, bull sharks have a lower deterrent threshold than white sharks (3 V/m *vs* 15.7 V/m)^10,13^, but also more ampullae of Lorenzini, and should theoretically be more sensitive to the electric pulses emitted by ESDs^17^. The apparent lower efficacy of the Freedom + Surf on bull sharks compared to white sharks raises questions on the relationship between the use and sensitivity of a species’ electrosensory system and their response to the electric pulses emitted by ESDs.

Conversely, the higher sensitivity of bull sharks to electric fields may explain the much higher proportion of aversive reactions to ESD pulses than other studies on the topic. The discrepancy may also be caused by the number of sharks approaching the board at once and affecting each other’s behaviours, as well as the lower effective distance between the sharks and the board in our experiment compared to previous papers. Sharks are more likely to exhibit a reaction to ESDs the closer they get to the board^13^, and bull sharks approached our experimental board at a much closer distance on average than white sharks^12^.

Bull sharks reacted to the ESD at a much shorter distance in our study, i.e. 40–50 cm, compared to white sharks, i.e. ~ 1.5 m (Huveneers et al. 2018). This discrepancy between studies might be linked to differences in visibility. In the present study, visibility was ~ 2–3 m, making it impossible to observe sharks reacting to ESDs from further away (if they occurred) and potentially biasing the average reaction distance, whereas the visibility in Huveneers et al. (2018) study consistently exceeded 10 m.

In contrast to results from Huveneers et al. (2018), two other deterrents significantly deterred sharks: Rpela v2 and Freedom + Surf—Shortboard. The Freedom + Surf—Shortboard uses the same power module and electric field characteristics as other Ocean Guardian products, confirming the efficacy of this type of electric pulses to deter sharks. The lower efficacy of the Freedom + Surf—Shortboard compared to the Freedom + Surf may be a consequence of trials on this device starting halfway through the trip, when sharks might have already started to become habituated to exposure to electric fields. The size of and distance between the Freedom + Surf—Shortboard electrodes are also smaller and closer together than the standard Freedom + Surf, which might have influenced its ability to deter bull sharks. This reinforces that the type of pulse emitted is not the only factor affecting the efficacy of ESDs, but that the size of and distance between electrodes and consequent field are also important. The Rpela v2 appears to more effectively reduce the likelihood of a bait being taken or touched than its predecessor^12^. The electric fields of the Rpela v2 covers a larger volume than the E-Shark Force and NoShark. However, the peculiar quasi-hemispherical shapes of each field around the electrodes, for an isosurface of 3 V/m, do not overlap between the electrodes where the bait was, likely contributing to a lower efficacy when compared to the Freedom + Surf. The Rpela v2 has smaller electrodes and fewer discharges per second (14.5 Hz *vs*. 9.5 Hz) than its predecessor, allowing the deterrent to better recharge between pulses and emit a stronger electric field^14^. The intensity of the electric field 1 m away from the electrode were previously measured as being over 1 V/m^15^, which would be comparable to the Freedom + Surf, but our in situ measurements revealed a lower intensity of 0.38 V/m.

Neither the E-Shark Force nor the NoShark statistically reduced the likelihood of the bait/board being touched/taken. However, bull sharks had significantly more reaction in proximity to these ESDs and had more passes before taking the bait than with the control board. As opposed to the Rpela v2 and Freedom + Surf the two electrodes of these ESDs are located on a bracelet and they do not possess the same range of protection. These two bracelet repellents also use a different pulse frequency and duration than the other ESDs tested in our study, emitting a large number of very fast electric pulses over ~ 2 s, before recharging for up to 2 s and starting the cycle again^27^. This potentially allows bull sharks to approach the bait more easily during the recharging period, compared to the more frequent pulses emitted by Ocean Guardian and Rpela products.

Our findings suggest some level of habituation, with bull shark responses to ESDs decreasing throughout the study. Although sharks continued to react to the ESD when within ~ 40 cm of the board, they came increasingly closer, became more likely to take the bait regardless of the active ESD, and did so in a smaller number of passes. Such habituation and decrease in the efficacy of an electromagnetic field has previously been observed in Galapagos, *Carcharhinus galapagensis*, sandbar, *Carcharhinus plumbeus*, lemon, *Negaprion brevirostris*, great hammerhead, *Sphyrna mokkaran*^22,28,30^ and on white sharks^13^. The latter showed that white sharks got closer to the bait canister and more likely to interact with it throughout the trials, similarly to bull sharks in our study^13^. Huveneers et al. (2018) observed no evidence of habituation. All our trials were realised in 18 days, over a 21-day period, and with fewer than 40 individual sharks. Trials in other studies were either done over 18 days spread between five trips over 5 months^12^, or during a short one-week trip on a large number of sharks^14^. During these studies, the same individual sharks were not as frequently exposed to electric fields as our study, reducing the ability to detect habituation or temporal changes in response to electric fields.

Four sharks were observed interacting with the board in more than fifteen trials, and together were responsible for over half of all baits taken. By Day 10, any of these four sharks consistently grabbed the bait in 1–2 passes, regardless of whether a deterrent was active. If we remove these four individuals from the study, the percentage of baits taken with an active Freedom + Surf would be reduced from 57.7 to about 43%, a reduction comparable to findings from Huveneers et al. (2018). However, there is evidence of a significant temporal effect without these four sharks, confirming that they were not the only ones exhibiting signs of habituation. This level of habituation is unlikely to occur in the context of swimmers and surfers using ESDs. In our study, individual bull sharks were frequently exposed electric fields within the 21-day study period (i.e. up to 227 passes and 127 trials). Such high frequency of exposure is unlikely to occur in natural conditions even if bull sharks reside in some areas for extended periods. Further studies on potential habituation or conditioning to electric fields would be necessary to unravel how long-term use of ESDs might affect shark behaviour.

The comparison and concordance between the in-situ measurements and theoretical modelling of the electric fields produced by the ESDs validate the use of the theoretical model for our analysis. The tri-dimensional analysis allows for a better visual representation of the effective range of each ESD with an electric field of 3 V/m as the repellent threshold for bull sharks^8^. Future work on the effectiveness of ESDs should include the position and trajectory of sharks in relation to the electrodes, similar to the analysis in Thiele et al. (2020), due to the anisotropy of the electric fields of all deterrents. Such analysis would allow for more accurate information on the strength of the electric fields the sharks came into contact with, which could either confirm or give a more accurate estimate than the 3 V/m repellent threshold used here.

Our findings suggest that the Freedom + Surf is currently the most effective deterrent for surfers and can reduce shark bite risk from bull sharks. However, bull sharks were still able to consume the sardine in some trials with any of the deterrents active, showing that none of the tested deterrents can stop bull sharks in all conditions. Our study also shows that even when deterred, bull sharks could still get very close to the board (within ~ 40 cm). Bull sharks seemed to become habituated to ESDs reducing their efficacy over time. The frequency of exposure experienced during this study is, however, unlikely to occur with swimmers and surfers. Overall, this study adds to the body of work showing electric fields as promising shark deterrents, but that their range is currently limited, potentially affecting their broader effectiveness.

## Supplementary information

Supplementary Information.

## References

[1] McPhee D (2014). Unprovoked shark bites: Are they becoming more prevalent?. Coast. Manage..

[2] Gallagher AJ (2015). Biological effects, conservation potential, and research priorities of shark diving tourism. Biol. Conserv..

[3] Midway SR, Wagner T, Burgess GH (2019). Trends in global shark attacks. PLoS ONE.

[4] Crossley R, Collins CM, Sutton SG, Huveneers C (2014). Public perception and understanding of shark attack mitigation measures in Australia. Hum. Dimens. Wildl..

[5] Myrick JG, Evans SD (2014). Do PSAs take a bite out of Shark Week? The effects of juxtaposing environmental messages with violent images of shark attacks. Sci. Commun..

[6] Muter BA, Gore ML, Gledhill KS, Lamont C, Huveneers C (2012). Australian and U.S. news media portrayal of sharks and their conservation. Conserv. Biol..

[7] Sabatier E, Huveneers C (2018). Changes in media portrayal of human-wildlife conflict during successive fatal shark bites. Conserv. Soc..

[8] West JG (2011). Changing patterns of shark attacks in Australian waters. Mar. Fresh. Res..

[9] Pepin-Neff C, Hueter R (2013). Science, policy, and the public discourse of shark “attack”: A proposal for reclassifying human-shark interactions. J. Environ. Stud. Sci..

[10] Dudley SFJ, Cliff G, Carrier JC, Musick JA, Heithaus MR (2010). Shark control: Methods, efficacy, and ecological impact. Sharks and their Relatives II: Biodiversity, Adaptive Physiology, and Conservation.

[11] Pepin-Neff C (2012). Australian beach safety and the politics of shark attacks. Coast. Manage..

[12] Huveneers C (2018). Effectiveness of five personal shark-bite deterrents for surfers. PeerJ.

[13] Kempster RM (2016). How close is too close? The effect of a non-lethal electric shark deterrent on white shark behaviour. PLoS ONE.

[14] Thiele M (2020). Response of blacktip reef sharks *Carcharhinus melanopterus* to shark bite mitigation products. Sci. Rep..

[15] Blount, C. RPELA v2—Testing effectiveness against white sharks. (2018).

[16] Egeberg CA (2019). Not all electric shark deterrents are made equal: Effects of a commercial electric anklet deterrent on white shark behaviour. PLoS ONE.

[17] Kempster RM, McCarthy ID, Collin SP (2012). Phylogenetic and ecological factors influencing the number and distribution of electroreceptors in elasmobranchs. J. Fish. Biol..

[18] Kalmijn A (1966). Electro-perception in sharks and rays. Nature.

[19] Marcotte MM, Lowe CG (2008). Behavioural responses of two species of sharks to pulsed direct current electrical fields: Testing a potential shark deterrent. Mar. Technol. Soc. J..

[20] Smit CF, Peddemors V (2003). Estimating the probability of a shark attack when using an electric repellent. S. Afr. Stat. J..

[21] Huveneers C (2013). Effects of an electric field on white sharks: In situ testing of an electric deterrent. PLoS ONE.

[22] Brill R (2009). The repulsive and feeding-deterrent effects of electropositive metals on juvenile sandbar sharks (*Carcharhinus plumbeus*). Fish. Bull..

[23] Kimber JA, Sims DW, Bellamy PH, Gill AB (2014). Elasmobranch cognitive ability: Using electroreceptive foraging behaviour to demonstrate learning, habituation and memory in a benthic shark. Anim. Cogn..

[24] Harvey E (2003). The accuracy and precision of underwater measurements of length and maximum body depth of southern bluefin tuna (*Thunnus maccoyii*) with a stereo-video camera system. Fish. Res..

[25] Burnham KP, Anderson DR (2002). Model Selection and Multimodel Inference: A Practical Information-Theoretic Approach.

[26] Fay MP (2010). Two-sided exact tests and matching confidence intervals for discrete data. R. J..

[27] Chateauminois, E., Hoarau, M., & Maillard, F. Résultats des tests expérimentaux sur les équipements de protection individuels répulsifs à impulsion électrique. (2019).

[28] Robbins WD, Peddemors VM, Kennelly SJ (2011). Assessment of permanent magnets and electropositive metals to reduce the line-based capture of Galapagos sharks, *Carcharhinus galapagensis*. Fish. Res..

[29] Brena PF, Mourier J, Planes S, Clua EE (2018). Concede or clash? Solitary sharks competing for food assess rivals to decide. Proc. R. Soc. B Biol. Sci..

[30] O’Connell CP, Hyun S-Y, Gruber SH, He P (2015). Effects of barium-ferrite permanent magnets on great hammerhead shark *Sphyrna mokarran* behaviour and implications for future conservation technologies. Endanger. Species Res..

