# Supplementary material for: Variable response to electric shark deterrents in bull sharks, Carcharhinus leucas
Source: Sci Rep. 2020 Oct 21;10:17869. doi: 10.1038/s41598-020-74799-y (PMC7578011; doi:10.1038/s41598-020-74799-y)
Supplement: Supplementary file 1 — Supplementary Information. [file 41598_2020_74799_MOESM1_ESM.docx]

**Variable response to electric shark deterrents in bull sharks, *Carcharhinus leucas***

Gauthier, A.R.G.^1^, Chateauminois, E.^1^, Hoarau, M.G.^1^, Gadenne, J.^1^, Hoarau, E.^1^, Jaquemet, S.^1,2^, Whitmarsh, S.K.^3^, Huveneers, C.^3^

1. Centre Sécurité Requin, 25F Avenue des Artisans, Zone Artisanale de la Pointe des Châteaux, 97436 Saint Leu, Reunion Island, France

2. Université de La Réunion UMR Entropie, 15, Avenue René Cassin - CS 92003, 97744 Saint Denis Cedex 9, Reunion Island, France

3. Southern Shark Ecology Group, College of Science and Engineering, Flinders University, Adelaide, Australia

**Corresponding author:** Arnault Gauthier, arnault.gauthier@securite-requin.re

**Keywords:** human-wildlife conflict, shark attack, shark bites, personal protective equipment, human protection, mitigation measures, habituation

**Supplementary materials.**

**Table S1.** Estimated deterrent level coefficients (β) and their standard errors (SE), z-values of factors included in the top-ranked model (indicated for each variable), and the individual coefficient Type I error estimate (P). The baseline factor is the control board.

| Level | β | SE | z | P |
| --- | --- | --- | --- | --- |
| Bait ~ deterrent + trial + deterrent*trial | | |  |  |
| intercept | -0.714 | 1.304 | -0.55 | 0.58 |
| E-Shark Force | -3.562 | 2.319 | -1.54 | 0.12 |
| Freedom+ Surf - Shortboard (Control) | -8.361 | 6.881 | -1.22 | 0.22 |
| Freedom+ Surf - Shortboard | -2.592 | 2.396 | -1.08 | 0.28 |
| No Shark | -0.515 | 1.471 | -0.35 | 0.73 |
| Rpela v2 | -0.248 | 1.419 | -1.18 | 0.86 |
| Freedom+ Surf | 2.886 | 1.432 | 2.02 | 0.04 |
| Trial | -0.174 | 0.133 | -1.31 | 0.19 |
| E-Shark Force*trial | 0.174 | 0.138 | 1.26 | 0.21 |
| Freedom+ Surf - Shortboard (Control)*trial | 0.233 | 0.149 | 1.56 | 0.12 |
| Freedom+ Surf - Shortboard*trial | 0.193 | 0.135 | 1.43 | 0.15 |
| No Shark*trial | 0.144 | 0.134 | 1.07 | 0.28 |
| Rpela v2*trial | 0.159 | 0.134 | 1.19 | 0.23 |
| Freedom+ Surf*trial | 0.108 | 0.134 | 0.81 | 0.42 |
|  |  |  |  |  |
|  |  |  |  |  |
|  |  |  |  |  |
| Distance ~ deterrent + trial + deterrent*trial | | |  |  |
| intercept | 2.894 | 0.098 | 29.42 | <0.01 |
| E-Shark Force | -0.036 | 0.128 | -0.28 | 0.78 |
| Freedom+ Surf - Shortboard (Control) | -0.359 | 0.442 | -0.81 | 0.42 |
| Freedom+ Surf - Shortboard | -0.284 | 0.212 | -1.34 | 0.18 |
| No Shark | 0.017 | 0.122 | 0.14 | 0.89 |
| Rpela v2 | -0.194 | 0.119 | -1.63 | 0.10 |
| Freedom+ Surf | -0.005 | 0.108 | -0.05 | 0.96 |
| Trial | -0.003 | 0.003 | -1.34 | 0.18 |
| E-Shark Force*trial | -0.003 | 0.004 | -0.78 | 0.44 |
| Freedom+ Surf - Shortboard (Control)*trial | -0.006 | 0.005 | 1.02 | 0.31 |
| Freedom+ Surf - Shortboard*trial | 0.003 | 0.003 | 0.72 | 0.47 |
| No Shark*trial | -0.004 | 0.003 | -1.14 | 0.26 |
| Rpela v2*trial | 0.005 | 0.003 | 1.44 | 0.15 |
| Freedom+ Surf*trial | -0.004 | 0.003 | -1.24 | 0.22 |
|  |  |  |  |  |
|  |  |  |  |  |
|  |  |  |  |  |
| Passes ~ deterrent + trial | | |  |  |
| intercept | -0.195 | 0.028 | -7.02 | <0.01 |
| E-Shark Force | 0.043 | 0.033 | 1.32 | 0.19 |
| Freedom+ Surf - Shortboard (Control) | 0.077 | 0.038 | 2.00 | 0.046 |
| Freedom+ Surf - Shortboard | 0.178 | 0.037 | 4.85 | <0.01 |
| No Shark | 0.067 | 0.032 | 2.10 | 0.04 |
| Rpela v2 | 0.105 | 0.032 | 3.26 | <0.01 |
| Freedom+ Surf | 0.185 | 0.032 | 5.76 | <0.01 |
| Trial | -0.002 | <0.001 | -5.84 | <0.01 |
|  |  |  |  |  |
|  |  |  |  |  |
|  |  |  |  |  |
| Reaction~deterrent |  |  |  |  |
| Intercept | -1.513 | 0.217 | -6.98 | <0.01 |
| E-Shark Force | 1.258 | 0.265 | 4.75 | <0.01 |
| Freedom+ Surf - Shortboard (Control) | -0.340 | 0.345 | -0.98 | 0.33 |
| Freedom+ Surf - Shortboard | 2.158 | 0.266 | 8.11 | <0.01 |
| NoShark | 1.704 | 0.259 | 6.57 | <0.01 |
| Rpela v2 | 1.852 | 0.253 | 7.31 | <0.01 |
| Freedom+ Surf | 2.853 | 0.259 | 11.00 | <0.01 |
|  |  |  |  |  |
|  |  |  |  |  |
|  |  |  |  |  |

**Table S2.** Estimated deterrent level coefficients (β) and their standard errors (SE), z-values of factors included in the top-ranked model (indicated for each variable), and the individual coefficient Type I error estimate (P) when relevant. All models include Shark ID as a random factor. The baseline factor is the control board.

| Level | β | SE | z | P |
| --- | --- | --- | --- | --- |
| Bait ~ deterrent + trial |  |  |  |  |
| intercept | -2.381 | 1.346 | -1.77 | 0.08 |
| E-Shark Force | -23.730 | 1.686 | 0.00 | 0.99 |
| Freedom+ Surf - Shortboard (Control) | -15.550 | 1.710 | <-0.01 | 0.99 |
| Freedom+ Surf - Shortboard | 3.697 | 1.864 | 1.98 | 0.047 |
| No Shark | 1.197 | 1.431 | 0.84 | 0.40 |
| Rpela v2 | 2.252 | 1.349 | 1.67 | 0.10 |
| Freedom+ Surf | 4.725 | 1.371 | 3.45 | <0.01 |
| Trial | -0.054 | 0.018 | -3.07 | <0.01 |
|  |  |  |  |  |
|  |  |  |  |  |
|  |  |  |  |  |
| Distance ~ deterrent + trial |  |  |  |  |
| intercept | 2.910 | 0.085 | 34.26 |  |
| E-Shark Force | -0.147 | 0.097 | -1.51 |  |
| Freedom+ Surf - Shortboard (Control) | 0.244 | 0.137 | 1.77 |  |
| Freedom+ Surf - Shortboard | -0.088 | 0.118 | -0.75 |  |
| No Shark | -0.100 | 0.093 | -1.07 |  |
| Rpela v2 | -0.094 | 0.087 | -1.08 |  |
| Freedom+ Surf | -0.132 | 0.085 | -1.55 |  |
| Trial | -0.001 | 0.001 | -4.85 |  |
|  |  |  |  |  |
|  |  |  |  |  |
|  |  |  |  |  |
| Passes ~ deterrent + trial |  |  |  |  |
| intercept | -0.126 | 0.031 | -4.05 |  |
| E-Shark Force | 0.016 | 0.036 | 0.44 |  |
| Freedom+ Surf - Shortboard (Control) | 0.020 | 0.041 | 0.47 |  |
| Freedom+ Surf - Shortboard | 0.089 | 0.040 | 2.22 |  |
| No Shark | 0.029 | 0.035 | 0.83 |  |
| Rpela v2 | 0.068 | 0.035 | 1.95 |  |
| Freedom+ Surf | 0.121 | 0.036 | 3.33 |  |
| Trial | -0.002 | <0.001 | -4.20 |  |
|  |  |  |  |  |
|  |  |  |  |  |
|  |  |  |  |  |
| Reaction~deterrent + trial |  |  |  |  |
| Intercept | -0.941 | 0.346 | -2.72 | <0.01 |
| E-Shark Force | 0.699 | 0.375 | 1.86 | 0.06 |
| Freedom+ Surf - Shortboard (Control) | -0.302 | 0.570 | -0.53 | 0.60 |
| Freedom+ Surf - Shortboard | 2.760 | 0.442 | 6.25 | <0.01 |
| No Shark | 1.689 | 0.357 | 4.73 | <0.01 |
| Rpela v2 | 1.820 | 0.351 | 5.19 | <0.01 |
| Freedom+ Surf | 3.511 | 0.407 | 8.63 | <0.01 |
| Trial | -0.006 | 0.004 | -1.47 | 0.14 |

**Table S3.** Summary of additional analyses performed. GLMs were performed on the dataset while separating out the Freedom+ Surf – Shortboard trials from the rest of the experiment, as well as separating the four sharks that interacted with the board more than 15 times. Finally, we created an additional factor, *Period*¸ to confirm the effects of *trial-set* on all response variables over time.

**
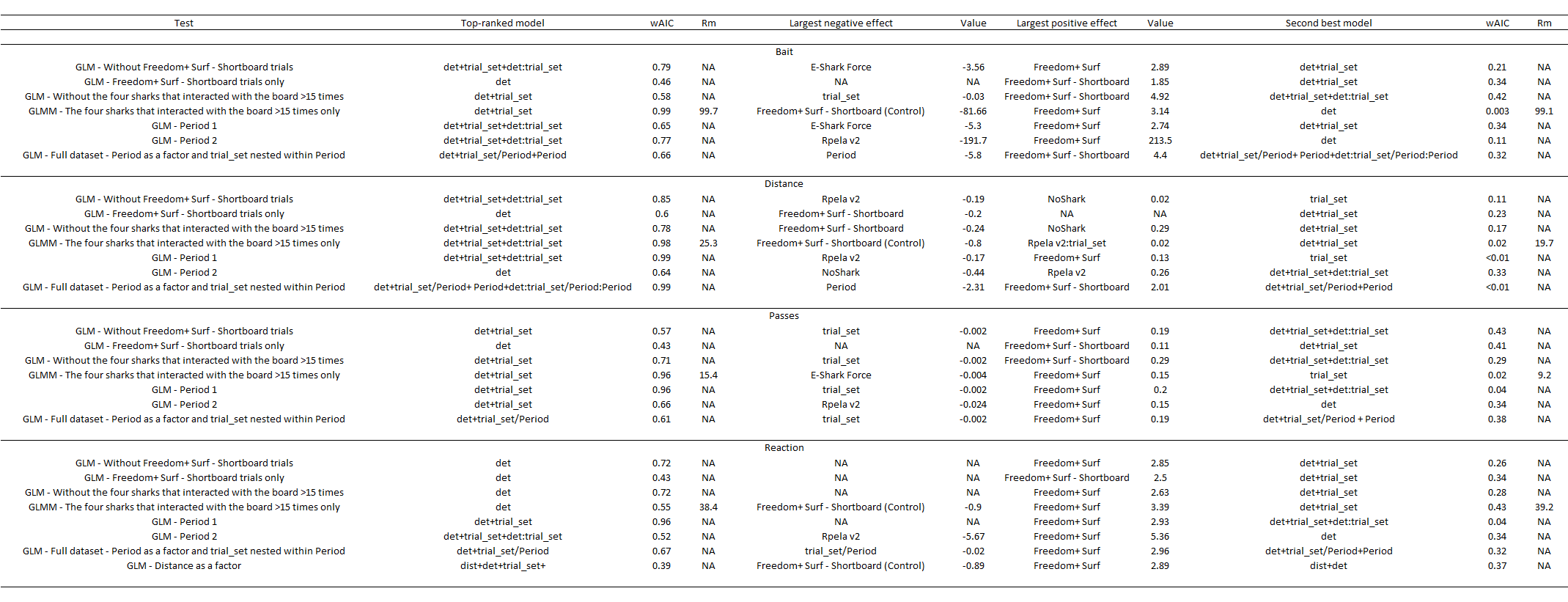
**


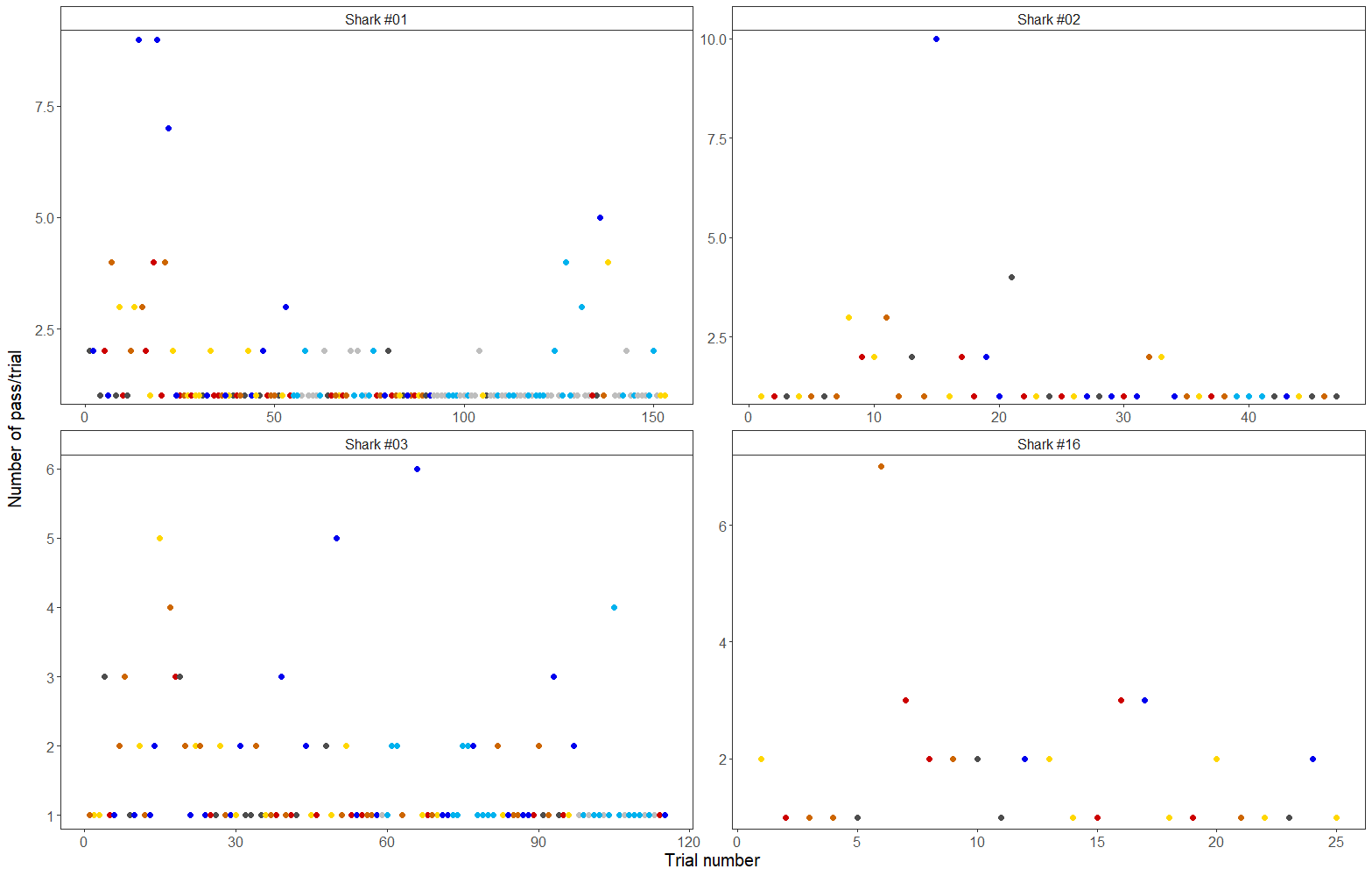


**Figure S1.** Numbers of passes during the 15-minute trials for the four sharks that interacted with the board on 15 trials or more. Dark grey: *Control*, red: *E-Shark Force*, yellow: *NoShark*, Orange: *Rpela v2*, dark blue: *Freedom+ Surf*, light grey: *Freedom+ Surf – Shortboard (Control)*, light blue: *Freedom+ Surf – Shortboard*.


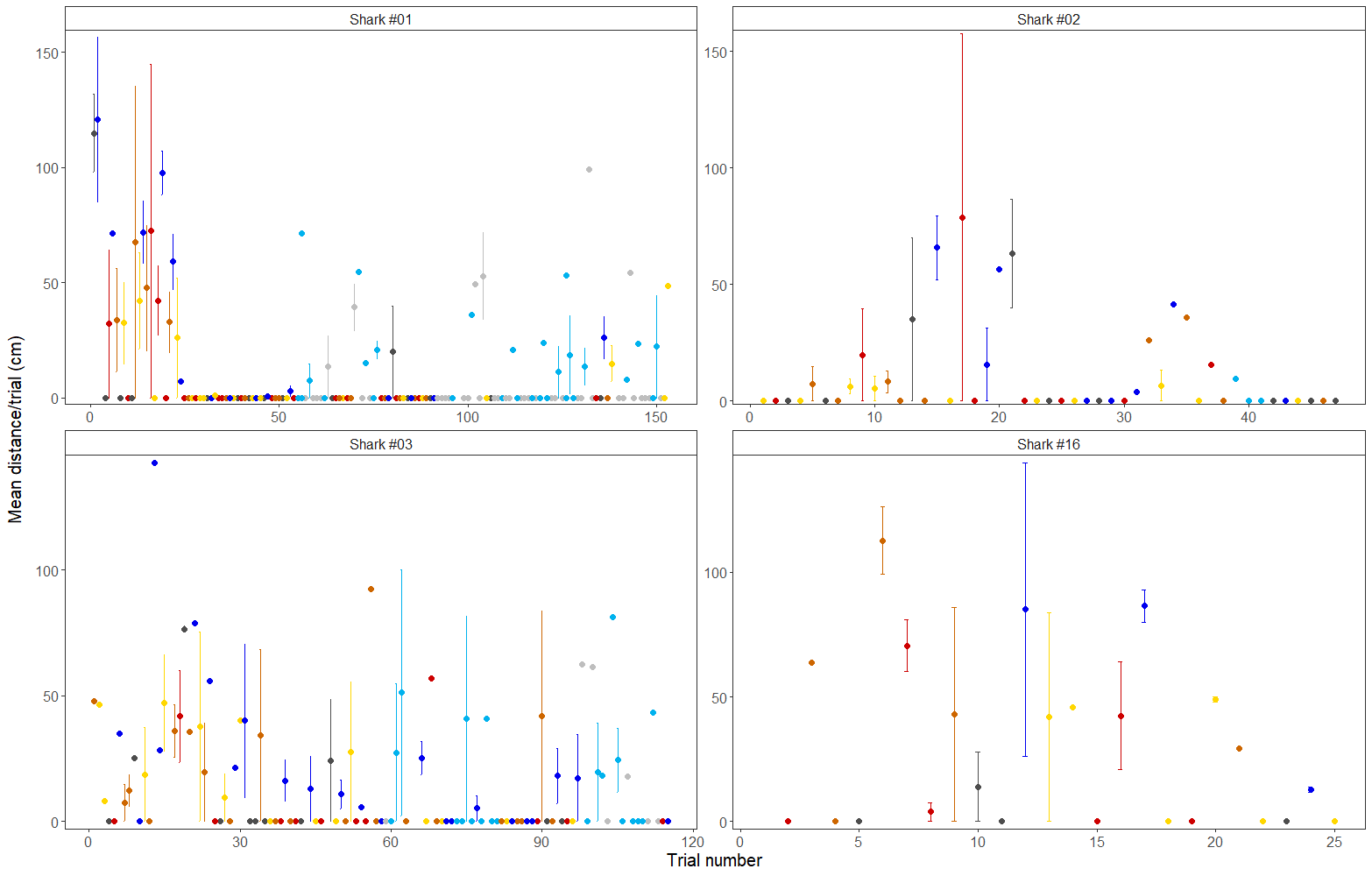


**Figure S2.** Mean distance between bull sharks and the bait for the four sharks that interacted with the board on 15 trials or more. Error bars represent standard errors. Dark grey: *Control*, red: *E-Shark Force*, yellow: *NoShark*, Orange: *Rpela v2*, dark blue: *Freedom+ Surf*, light grey: *Freedom+ Surf – Shortboard (Control)*, light blue: *Freedom+ Surf – Shortboard*.


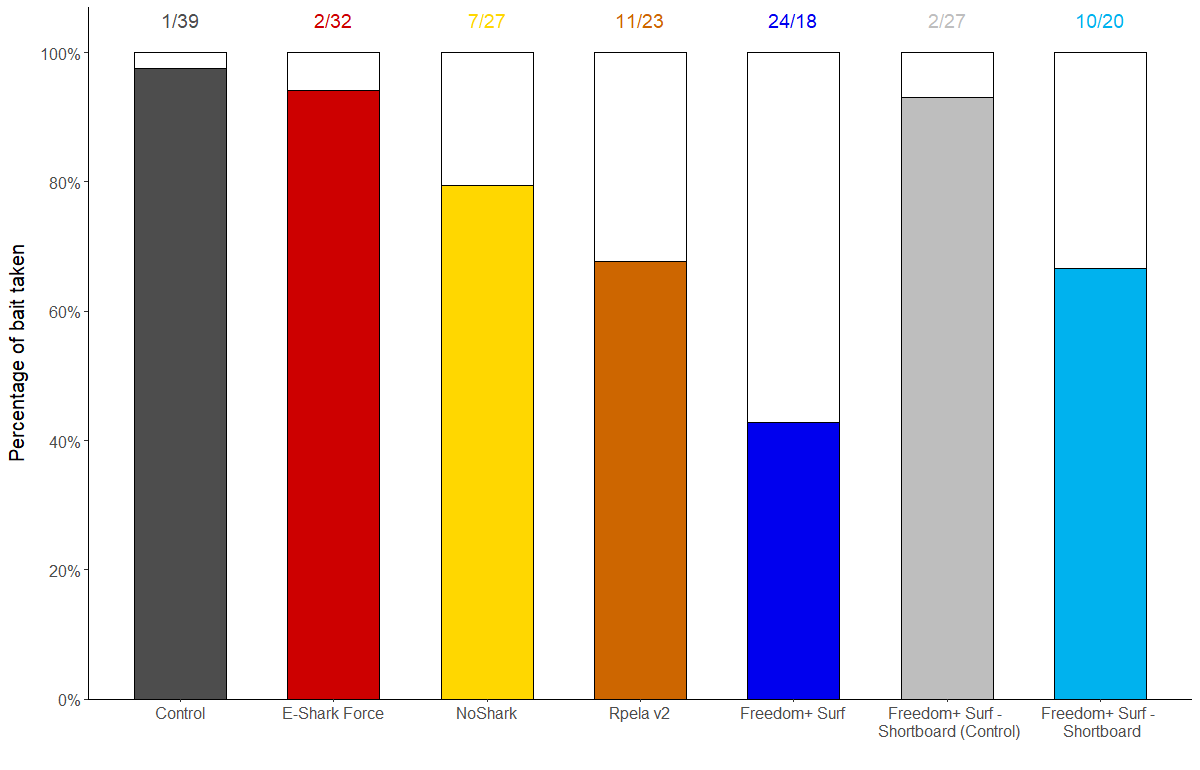


**Figure S3.** Effects of ESDs on the percentage of baits taken by bull sharks during trials, with the four sharks (#01, #02, #03, #16) that interacted with the board on more than 15 trials removed. White bars represent trials when the board or bait were not touched or taken. Numbers above the bars represent the number of trials with board or bait not touched/taken or touched/taken.
